# Rényi entropy-complexity causality space: a novel neurocomputational tool for detecting scale-free features in EEG/iEEG data

**DOI:** 10.3389/fncom.2024.1342985

**Published:** 2024-07-15

**Authors:** Natalí Guisande, Fernando Montani

**Affiliations:** Instituto de Física de La Plata (IFLP), Consejo Nacional de Investigaciones Científicas y Técnicas – Universidad Nacional de La Plata (CONICET-UNLP), La Plata, Buenos Aires, Argentina

**Keywords:** scale-free brain activity, neuronal encoding, Rényi entropy-complexity causality space, EEG/iEEG signals, information theory, brain dynamics, power law, permutation entropy

## Abstract

Scale-free brain activity, linked with learning, the integration of different time scales, and the formation of mental models, is correlated with a metastable cognitive basis. The spectral slope, a key aspect of scale-free dynamics, was proposed as a potential indicator to distinguish between different sleep stages. Studies suggest that brain networks maintain a consistent scale-free structure across wakefulness, anesthesia, and recovery. Although differences in anesthetic sensitivity between the sexes are recognized, these variations are not evident in clinical electroencephalographic recordings of the cortex. Recently, changes in the slope of the power law exponent of neural activity were found to correlate with changes in Rényi entropy, an extended concept of Shannon's information entropy. These findings establish quantifiers as a promising tool for the study of scale-free dynamics in the brain. Our study presents a novel visual representation called the Rényi entropy-complexity causality space, which encapsulates complexity, permutation entropy, and the Rényi parameter q. The main goal of this study is to define this space for classical dynamical systems within theoretical bounds. In addition, the study aims to investigate how well different time series mimicking scale-free activity can be discriminated. Finally, this tool is used to detect dynamic features in intracranial electroencephalography (iEEG) signals. To achieve these goals, the study implementse the Bandt and Pompe method for ordinal patterns. In this process, each signal is associated with a probability distribution, and the causal measures of Rényi entropy and complexity are computed based on the parameter q. This method is a valuable tool for analyzing simulated time series. It effectively distinguishes elements of correlated noise and provides a straightforward means of examining differences in behaviors, characteristics, and classifications. For the iEEG experimental data, the REM state showed a greater number of significant sex-based differences, while the supramarginal gyrus region showed the most variation across different modes and analyzes. Exploring scale-free brain activity with this framework could provide valuable insights into cognition and neurological disorders. The results may have implications for understanding differences in brain function between the sexes and their possible relevance to neurological disorders.

## 1 Introduction

In brain activity, oscillations and scale-free neuronal activity coexist (Zilber et al., 2013; He, 2014; Zilber, 2014; Bongers et al., 2020). Brain oscillations are recurrent patterns of neuronal activity that follow a specific temporal rhythm. In the study of brain electrical activity, different frequency bands have been identified, each with its own characteristics and correlations with specific cognitive states and brain functions (He, 2014).

Regardless of its precise origin, across various spatiotemporal scales, brain activity exhibits a power spectrum that conforms to a *f*^−*k*^ power law, as evidenced by local field potentials (LFPs; Miller et al., 2009; He et al., 2010; Jones et al., 2023). This implies that as the frequency increases, the power decreases, suggesting intricate self-organization and self-regulation within the brain across different levels (Marković and Gros, 2014; Plenz et al., 2021; Grosu et al., 2022). A power law function signifies scale invariance, indicating that no specific time or frequency scale dominates the dynamics, resulting in an absence of periodicity (He, 2014).

For decades, scale-free brain activity, a type of brain activity without dominant frequencies, has been considered unimportant and often dismissed as background noise. On many occasions, it was excluded from analyzes to emphasize brain oscillations. However, there is growing evidence suggesting that both brain oscillations and aperiodic brain activity exist, and the latter actively contributes to brain functioning (He, 2014; Grosu et al., 2022; Jones et al., 2023). The existence of scale-free brain activity has been associated with the learning process, suggesting its potential importance in integrating different temporal scales and shaping cognitive frameworks (Zilber et al., 2013; Zilber, 2014; Bongers et al., 2020). Similar behaviors have been investigated in speech, linking them to the metastable foundation of cognition (Kello et al., 2008).

That is, neurophysiological signals are partly characterized by non-oscillatory activity consistent with a *f*^−*k*^ pattern. Under the oscillatory peaks, the “background” of the power spectral density (PSD) exhibits a decay from slower to faster frequencies, following an inverse power law distribution, resembling the shape of a *f*^−*k*^ curve. The underlying source of this background activity may stem from either truly irregular patterns of neuronal firing (Juel et al., 2018) or from brief oscillations with varying frequencies observed across a wide spatial or temporal range (Palva and Palva, 2018). This phenomenon is evident in diverse neural signals, including electrocorticography (He et al., 2010), local field potentials (Buzsáki and Mizuseki, 2014), membrane potential fluctuations (Destexhe et al., 2003), functional magnetic resonance imaging (He, 2011), and magnetoencephalography (Dehghani et al., 2010). The scale-free distribution of brain activity leads to variations in the power law exponent that are typical of different functional neurophysiological states (He, 2014; Tozzi et al., 2018). Importantly, recent research has explored the relationship between changes in scaling slope and changes in Rényi entropy, which is an extension of Shannon's information entropy (Tozzi et al., 2018).

In information theory, it is common to use Shannon permutation entropy to distinguish among time series. However, in some cases, comparing permutation entropy alone is inadequate to differentiate time series exhibiting regular, chaotic, and stochastic behaviors (Rosso et al., 2007). Time series associated with the fully developed chaos of the logistic map and time series associated with correlated power law noise can exhibit nearly identical permutation entropy values (Rosso et al., 2007). For this reason, it is common to use both permutation entropy and another measure of complexity known as statistical complexity simultaneously (López-Ruiz et al., 1995; Anteneodo and Plastino, 1996; Lamberti et al., 2004). Statistical complexity is essentially the product of the normalized permutation entropy and the difference in probability distributions between the ordinal pattern probability distribution and the uniform distribution (Jauregui et al., 2018).

The values of Shannon entropy (*H*) and statistical complexity (*C*) associated with a time series are often represented graphically as points (*H, C*) on the complexity-entropy causality plane (*H*×*C*), where the term “causality” refers to the consideration of temporal correlations in the Bandt and Pompe approach (Bandt and Pompe, 2002; Rosso et al., 2007; Pessa and Ribeiro, 2021).

In time series of both chaotic and stochastic nature, noise can be distinguished from chaos as they are separated in this plane. This separation enables the differentiation between noise and chaos (Rosso et al., 2007; Montani and Rosso, 2014).

Recently, there have been descriptions of several situations where entropy and complexity values alone were insufficient to distinguish between time series of different natures (Ribeiro et al., 2017). This has led to the extension of these concepts from the causal entropy-complexity plane to generalized entropies such as Tsallis (Ribeiro et al., 2017) and Rényi (Jauregui et al., 2018). By combining Rényi entropy with a generalized form of statistical complexity, Jauregui et al. associated a parametric curve (the Rényi complexity-entropy curve) with a given time series (Jauregui et al., 2018). This approach demonstrates that these curves effectively distinguish between chaotic, stochastic, and periodic time series (Jauregui et al., 2018), with the parameter *q* playing a crucial role.

The *f*^−*k*^ behavior is observed across various brain states, encompassing both pathological and physiological conditions (Colombo et al., 2019; Medel et al., 2023). While unconsciousness is associated with EEG slowing (a shift in EEG power spectral distribution (PSD) from higher to lower frequencies), it's important to note that these oscillations are not exclusive indicators of an unconscious state (Colombo et al., 2019). Recent evidence suggests that both complexity and power law decay serve as key indicators of these states (Medel et al., 2023).

Research shows that brain networks maintain a scale-free global organization during consciousness, anesthesia, and recovery phases (Lee et al., 2010). Anesthetics play a crucial role in millions of life-saving treatments performed while patients are unconscious (Wasilczuk et al., 2024). Although sex differences in sensitivity to anesthetics are well-documented, with sex hormones playing a fundamental role in modulating sensitivity (Braithwaite et al., 2023; Wasilczuk et al., 2024), these differences are not discernible in the cortical electroencephalographic records commonly used in clinical settings (Wasilczuk et al., 2024).

These findings highlight the critical need to examine the influence of scale-free activity on neuronal encoding, particularly in the areas of learning, memory, and different states of consciousness. Fundamentally, it is scientifically relevant to distinguish scale-free brain activity among biological sexes, as it could contribute to elucidating, for example, the origin of clinical differences in response to anesthesia between men and women.

Motivated by the procedure of Jauregui et al. (2018), our current study proposes the implementation of Rényi causal entropy (*H*_*q*_; Rényi, 1961; Pessa and Ribeiro, 2021) and associated complexity (*C*_*q*_; Martin et al., 2006) to calculate multiple causal entropy-complexity planes that form a space for analyzing the scale-free characteristics of brain signals. These planes are depicted as functions of the Rényi parameter (*q*), which, when combined, create what is referred to as the Rényi entropy-complexity causality space (*H*_*q*_×*C*_*q*_×*q*). This space adeptly distinguishes the complexity-entropy curves of various systems, including those that obey power laws, even in situations where their projections onto a plane overlap. Both theoretical and experimental cases are examined in this work.

Through a simple simulation, we modeled scale-free brain dynamics using time series of correlated noise, which are then analyzed within the Rényi entropy-complexity causality space. To demonstrate its applicability to real-world datasets, this methodology is applied to iEEG data from the Montreal Neurological Institute (MNI) atlas (Frauscher et al., 2018a,b; von Ellenrieder et al., 2020), covering different brain regions, to investigate disparities in neural dynamics associated with biological sex. This application was chosen based on a large body of evidence highlighting differences in both structural and dynamic aspects of the brain between individuals of different biological sexes (Silas et al., 2010; Arnegard et al., 2020; Bučková et al., 2020; Cave and Barry, 2021).

In addition, the $\frac{1}{f}$ spectral slope of the EEG, which indicates scale-free activity, has been proposed as an arousal marker to distinguish between different states of wakefulness and sleep (Kozhemiako et al., 2022; Schneider et al., 2022). Specifically, differences between men and women have been noted, especially during REM sleep, with men generally having flatter slopes than women in all states (Kozhemiako et al., 2022). Our methodology aims to identify and visualize these differences within the Rényi entropy-complexity causality space.

The primary objective of this study is to introduce the Rényi entropy-complexity causality space as an innovative computational tool useful for identifying differences attributed to scale-free behavior. This tool aims to streamline the study of scale-free dynamics in the brain, allowing for precise differentiation of different neural dynamical features. Specifically, we aim to evaluate how classical dynamical systems are positioned within this space and to apply the computational tool to simulated time series and experimental data. The goal of this effort is to identify differences in neural dynamical properties that are due to scale-free behavior and to improve the understanding and analysis of complex brain dynamics, especially those that exhibit scale-free patterns.

## 2 Methods

### 2.1 Calculating time-causal quantifiers in information theory

This section outlines the methodologies employed to compute the causal theoretical quantifiers for the LFPs acquired from iEEG. Accurately quantifying the information content within observed neural activity is vital for the analysis of neural systems. Integrating “permutation patterns” with various metrics offers a more comprehensive understanding of the characteristics of a time series (Olivares et al., 2020).

The initial step in quantifying information using causal entropy measures for a time series involves associating it with a probability distribution function (PDF) extracted using the Bandt and Pompe (BP) methodology. Each time series, χ(*t*) = {*x*_*t*_; *t* = 1, ⋯ , *M*}, consists of *M* measurements of the observable χ. To analyze the time series, it is divided into *n* = *M*−(*D*−1)τ overlapping segments, where the chosen embedding dimension is *D* and the embedding delay is τ. This method is based on the construction of a histogram of ordinal patterns. A brief description is given here, as it has been studied extensively, but the reader is referred to the following references for a formal explanation, simple examples, and similar applications (Bandt and Pompe, 2002; Jauregui et al., 2018; Pessa and Ribeiro, 2021; Zanin and Olivares, 2021; Amigó and Rosso, 2023; Guisande et al., 2023).

The partitions are represented by a *D*-dimensional vector, which is used to determine the permutation of index numbers. All possible permutations of order *D* are considered, and the relative frequency of each permutation is calculated to obtain the distribution of ordinal patterns. Thus, the frequency of BP can be calculated using the following equation:


$$\begin{array}{l} p_{j}\left({\text{Π}}_{j}\right)=\frac{\text{number of partitions of type }{\text{Π}}_{j}\text{ in }\pi_{i}}{n}\;, \end{array}$$

where *p*_*j*_(Π_*j*_) represents the relative frequency of the *j*−*th* ordinal pattern, Π_*j*_, and π_*i*_ is the sequence of all ordinal patterns for all partitions. Note that the estimated PDF is discrete since it is computed using a histogram. It is also important to emphasize that for reliable statistics, the analyzed time series, χ(*t*), needs to be much longer than the total number of possible ordinal patterns (*M* >> *D*!. where *D*! represents the total number of possible ordinal patterns of size *D*).

This study employs the method of ordinal patterns to identify and quantify the existence of ordinal structures in time series data. The objective is to compare computationally simulated data, based on the power law (k-noise) and experimental data by analyzing the dynamics of physiological signals in normal brain regions across biological sexes, while taking into account the causality of the signals. Using the resulting probability distribution, the causal entropy (also known as permutation entropy) and causal complexity (also known as permutation complexity) of Rényi are computed. In this study, any mention of entropy or complexity refers specifically to these two quantifiers.

### 2.2 Rényi permutation entropy and Rényi statistical complexity

The Rényi entropy serves as a measure of uncertainty within a probability distribution. It offers a unique advantage over Shannon entropy, especially in the context of evaluating brain activity, due to its exceptional versatility in capturing diversity within complex systems. While Shannon entropy provides a single information index, Rényi entropy employs a parametric family of indices, allowing for a more comprehensive sensitivity to both rare and abundant elements (Jost, 2007).

The Rényi entropy is defined as (Rényi, 1961; Pessa and Ribeiro, 2021):


$$\begin{array}{l} S_{q}=\frac{1}{1-q}\ln\overset{N}{\underset{j=1}{\sum }}{p_{j}}^{q}\text{ for }q>0\wedge q\neq 1. \end{array}$$

In the context of BP, *N* represents the total number of possible states in the probability distribution, corresponding to the number of potential ordinal patterns (*N* = *D*!).

The *q* factor in the Rényi entropy formula functions as a weighting coefficient for the probability distribution. It indicates the order of entropy and determines the specific type of Rényi entropy being calculated. By adjusting the value of *q*, the Rényi entropy formula can reveal various aspects of the distribution, such as inclination toward rare events or focus on common events. Essentially, *q* facilitates the analysis of different characteristics of the probability distribution based on its value.

If the probabilities are uniform, all Rényi entropies of the distribution are equal, given by *S*_*q*_ = ln *N*. For non-uniform distributions, the entropies weakly decrease with respect to *q*. As *q* approaches 0, it converges to the max-entropy (*S*_*q* → 0_ = ln *N*), assigning equal weight to all possible events regardless of their probabilities. When *q* = 0, it represents the logarithm of the size of the support of χ. As *q* approaches ∞, it converges to the min-entropy $\left(S_{q\to \infty }=-\ln\;\max_{j}\left(p_{j}\right)\right)$, considering only events with the highest probability. The intermediate case at *q* = 1 corresponds to Shannon entropy $\left(S_{q=1}=-\overset{N}{\underset{j=1}{\sum }}p_{j}\ln\left(p_{j}\right)\right)$; (Zmeskal et al., 2013; Zhou and Zheng, 2022).

The adaptability of Rényi entropy makes it a useful measure for quantifying various levels of information and capturing the dynamics of non-stationary processes. By utilizing ordinal patterns, it enables the tracking of changes in entropy distribution over time (A-iyeh and Peters, 2016; Shalymov and Fradkov, 2016; Jauregui et al., 2018). Furthermore, Rényi entropy proves highly effective in describing multifractal systems (Jizba and Arimitsu, 2001) and maintains a close relationship with the scale-free exponent, making it a valuable tool for investigating the scale-free dynamics of the brain.

The human brain, with its intricate network of interconnected neurons, exhibits multifractal characteristics across various spatial and temporal scales (França et al., 2018). Hence, one of the primary reasons for selecting Rényi's entropic quantifiers as a central component of this approach is their effectiveness in describing multifractal systems. This approach can provide valuable insights into brain systems across different spatial and temporal scales, as well as varying levels of complexity (Tozzi et al., 2018).

Following the methodology of Martin et al. (2006), the Rényi statistical complexity is defined as:


$$\begin{array}{l} C_{q}=\frac{D_{q}\cdot H_{q}}{D_{q}^{*}}, \end{array}$$

where *H*_*q*_ is defined as


$$\begin{array}{l} H_{q}=\frac{S_{q}}{\ln\;N}. \end{array}$$

It is important to note that *D*_*q*_ (Jensen-Rényi divergence) is determined by


$$\begin{array}{l} D_{q}=\frac{1}{2(q-1)}\cdot (\ln\sum_{j=1}^{N}p_{j}^{q}{\left(\frac{p_{j}+\frac{1}{N}}{2}\right)}^{1-q}\\ \;+\ln\sum_{j=1}^{N}\frac{1}{N^{q}}{\left(\frac{p_{j}+\frac{1}{N}}{2}\right)}^{1-q}), \end{array}$$

and $D_{q}^{*}$ (a constant normalization representing the maximum value of *D*_*q*_) is given by


$$\begin{array}{l} D_{q}^{*}=\frac{1}{2\left(q-1\right)}\cdot \\ \;\ln\left(\frac{{\left(N+1\right)}^{1-q}+N-1}{N}{\left(\frac{N+1}{4N}\right)}^{1-q}\right). \end{array}$$

### 2.3 Modeling scale-free brain dynamics: generation and analysis of *k*-noise time series

To model and analyze patterns of brain activity exhibiting scale-free structures, correlated noise (k-noise) time series following a power law distribution (*f*^−*k*^) were computationally simulated. These time series simulate the intrinsic dynamics of scale-free neuronal activity, allowing modeling of the diverse and evolving nature of the brain across different time and frequency scales.

The computational generation process involved three essential steps: First, a set of *k* values representing the exponent of the power law distribution was defined using a logarithmic scale. Random noise data points were then generated and transformed from the time domain to the frequency domain using the Fast Fourier transform (FFT). The FFT decomposed the signal into its frequency components, which were then multiplied by a function that followed a specific power law distribution. This operation adjusted the amplitudes of different frequencies to match the desired power law distribution. Finally, the inverse Fourier transform was applied to obtain the resulting time series with power law characteristics.

After generating the time series, the BP methodology was used to associate these data points with a PDF. Subsequently, quantitative measures such as Rényi entropy and Rényi complexity were calculated for analysis purposes.

### 2.4 Dataset description: the atlas of the normal intracranial electroencephalogram

The time series corresponding to the LFPs utilized in this study were sourced from the MNI Open iEEG Atlas database. This database comprises recordings of intracranial activity in typical brain regions during various states, including quiet wakefulness with eyes closed (W), non-REM sleep stage N2 (N2), non-REM sleep stage N3 (N3), and REM sleep (R; Frauscher et al., 2018a,b; von Ellenrieder et al., 2020).

A total of 106 patients with focal epilepsy were included in the atlas, and recordings were made from 1,772 channels. Only channels located in gray matter and considered “normal” (far from epileptic regions) were utilized. Various types of intracerebral electrodes were employed, including Dixi, homemade MNI, and AdTech electrodes, as well as AdTech subdural strips and grids. Importantly, all recorded signals from these electrodes were incorporated into the analysis in this work without any differentiation between them.

The dataset contains patient information, including sex, channel type, hemisphere, channel name, channel position, and channel region. Additionally, all signals were resampled to 200 samples per second to ensure consistency. Power-line interference was minimized using an adaptive filter. Furthermore, all channels were zero-padded to a length of 68 s (13,600 samples) to maintain uniformity across segments, regardless of their number.

To facilitate the comparison of patient activity and the accumulation of results from multiple subjects, the electrodes were placed in a common stereotactic space. For more detailed information about the database and acquisition methods, readers can refer to the following references (Frauscher et al., 2018a,b; von Ellenrieder et al., 2020).

The signals were classified based on hemisphere, region, and biological sex, allowing for the analysis of each region in both hemispheres separately for female and male patients. To ensure a sufficient sample size for comparing behavior between males and females within each region, a criterion was established, requiring a minimum of five patients of each sex. This criterion was consistently applied, regardless of the type of electrodes used in the signals. Cases that did not meet this minimum requirement were excluded from the analysis.

To mitigate the effect of age, five males and five females were selected from each region of interest, specifically those that minimized differences in mean age and standard deviation between groups. The analysis was limited to the left hemisphere as it met more criteria in this region.

The LFPs from each channel for selected patients were analyzed, associating them with a PDF using the BP method. Subsequently, *H*_*q*_ and *C*_*q*_ were computed for *q* in the range [0.1, 7] with a step size of 0.01. The results for males and females were separately averaged within each region, and the standard deviation was calculated.

**Figure 1:**
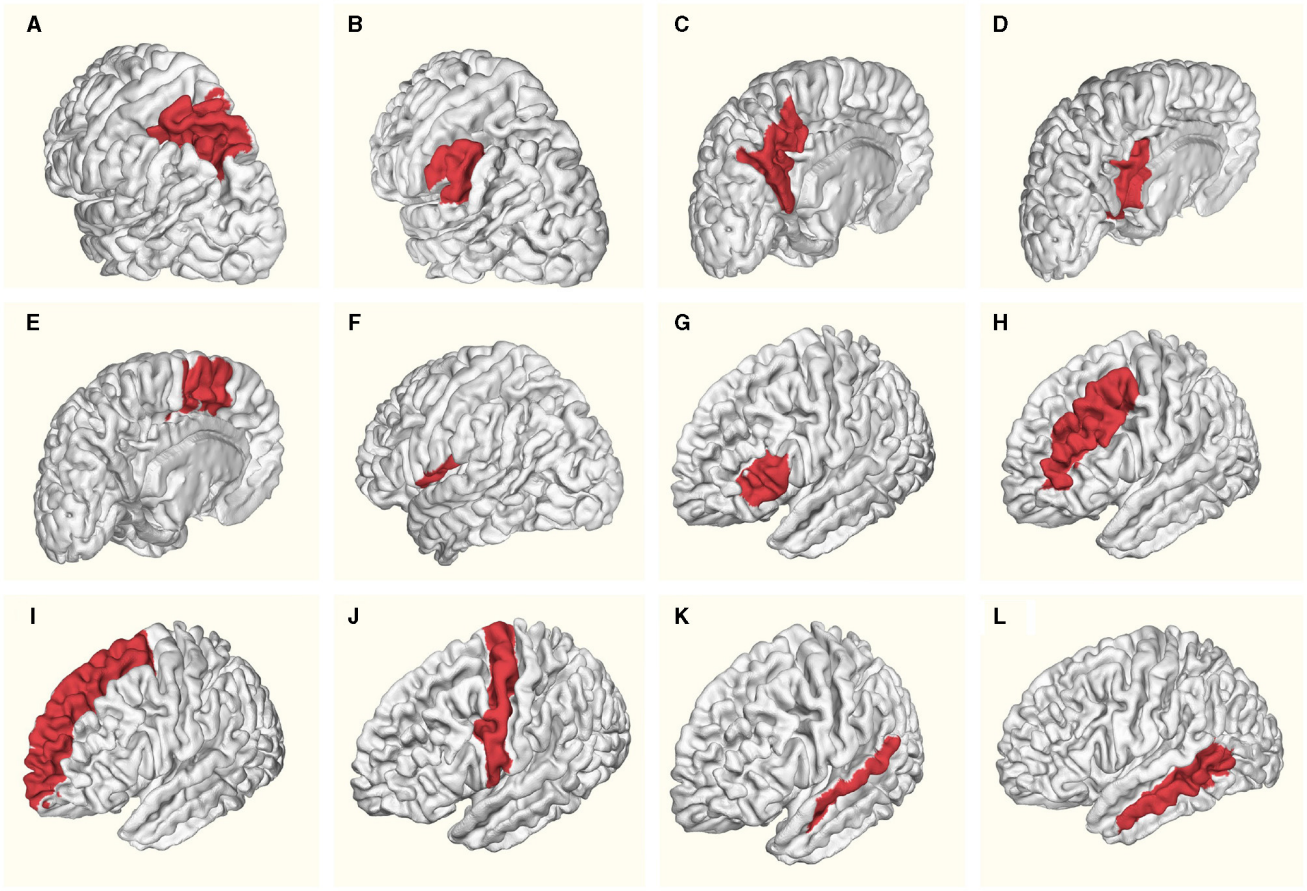
Regions of Interest (ROIs) identified in the left hemisphere: **(A)** Superior parietal lobule, **(B)** Supramarginal gyrus, **(C)** Precuneus, **(D)** Posterior cingulate, **(E)** Supplementary motor cortex, **(F)** Central operculum, **(G)** Triangular part of inferior frontal gyrus, **(H)** Middle frontal gyrus, **(I)** Superior frontal gyrus and frontal pole, **(J)** Precentral gyrus, **(K)** Superior temporal gyrus, **(L)** Middle temporal gyrus. Red shading indicates the specific region studied for each case. All highlighted areas belong to the left hemisphere.

The Table 1 provides a detailed overview of mean ages and standard deviation values for specific brain regions in both females and males within the left hemisphere. Figure 1 schematically illustrates the identified Regions of Interest (ROIs) in the left hemisphere. The red shading indicates the specific region studied for each case. All highlighted areas belong to the left hemisphere. This letter nomenclature for the ROIs is consistent throughout the study and is used in subsequent figures where the results for each region are analyzed. These ROIs were reconstructed using the nodes provided by the MNI Open iEEG Atlas, which follows the Desikan-Killiany parcellation.

**Table 1:** Mean ages (in years) of patients selected to minimize the differences of mean values between males and females in each analyzed region, along with their respective standard deviations.

**Left Hemisphere**				
**Region**	**Female**		**Male**	
	**Mean**	**Std**	**Mean**	**Std**
(A) Superior parietal lobule	29	10	41	16
(B) Supramarginal gyrus	27	7	32	10
(C) Precuneus	26	9	29	8
(D) Posterior cingulate	28	11	38	15
(E) Supplementary motor cortex	29	7	33	10
(F) Central operculum	24	6	31	8
(G) Triangular part of inferior frontal gyrus	34	8	36	5
(H) Middle frontal gyrus	33	9	33	4
(I) Superior frontal gyrus and frontal pole	26	7	34	6
(J) Precentral gyrus	26	6	26	6
(K) Superior temporal gyrus	36	12	36	4
(L) Middle temporal gyrus	37	5	37	4

**Figure 2:**
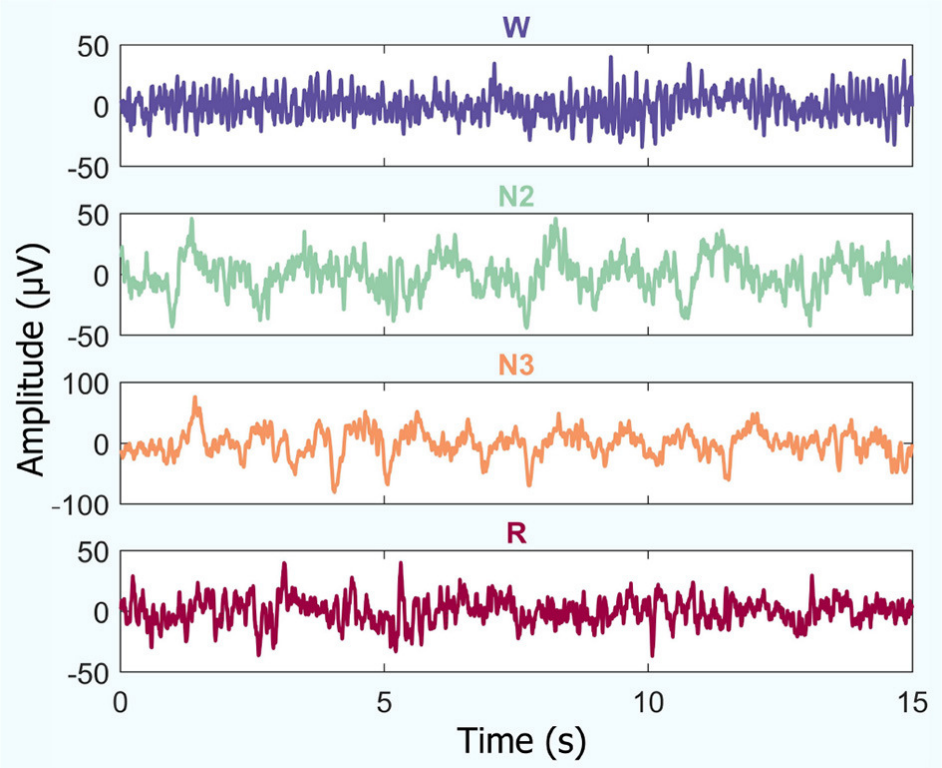
Temporal iEEG fragments depicting different brain states (for a random patient): amplitude vs. time. This includes periods of quiet wakefulness with eyes closed (W), non-REM sleep (stages N2 and N3), and REM sleep (R).

An example of temporal iEEG series fragments used to analyze the different brain states studied is shown in Figure 2.

### 2.5 Selection of embedding dimension, time delay, and Rényi parameter in analysis

An embedding dimension *D* = 3, 4, 5, and 6 for BP was chosen to investigate how the choice of embedding dimension affects the results. This selection satisfies the condition *M*≫*D*!, where the length of the atlas iEEG data was *M* = 13600.

In this study, the delay time of τ = 1, as recommended by Bandt and Pompe in their original article (Bandt and Pompe, 2002), was used to analyze causal relationships on a small time scale for theoretical scenarios.

For the analysis of iEEG, two time delays were implemented for each mode in the construction of the probability functions. On one hand, τ = 1 was implemented, aligning with the traditional BP methodology. On the other hand, previous studies have demonstrated a strong relationship between the parameter τ and the intrinsic temporal scales of the analyzed system (Soriano et al., 2011; Zunino et al., 2012, 2022). Hence, the embedding time (τ) was set equal to the shorter characteristic time (τ_*s*_) to account for the short intrinsic temporal scales of each mode. Although investigating different values of τ might have provided additional insights, the primary goal of this study was to match this parameter with the shorter characteristic time (τ_*s*_) observed in the analyzed iEEG and to maintain the traditional delay of τ = 1.

A reliable determination of a system's time delay can be achieved through permutation entropy and statistical complexity. Importantly, these metrics reach extremes when the embedding delay τ aligns with the characteristic delay τ_*s*_ of the system. The detection of this parameter is more sensitive using statistical complexity (Zunino et al., 2010a,b). The statistical complexity (MPR; Lamberti et al., 2004; Martin et al., 2006) was computed for the parameter τ, ranging from 1 to 30, with dimensions *D* set to 3, 4, 5, and 6, across all channels of the atlas in all states. The analysis showed that the highest value is achieved when τ = 1. Statistical complexity may be computed using:


$$C=Q_{J}\cdot H.$$

The normalized Shannon entropy *H* is defined as:


$$H=\frac{S}{S_{\text{max}}},$$

where *S*_max_ = *S*[*P*_*e*_] = log_2_*N*, and


$$S=-\overset{N}{\underset{j=1}{\sum }}p_{j}\log_{2}\left(p_{j}\right),$$

where *N* is the number of possible states of the physical system under consideration, and *p*_*j*_ is the probability of each state. The disequilibrium, $Q_{J}=Q_{0}J$, is determined by the Jensen-Shannon divergence, given by:


$$J=H\left[\frac{P+P_{e}}{2}\right]-\frac{H\left[P\right]}{2}-\frac{H\left[P_{e}\right]}{2},$$

with a normalization constant *Q*_0_. Here, *P*_*e*_ represents the uniform distribution $\left(P_{e}=\frac{1}{N}\right)$. To obtain the statistical complexity, $Q_{J}$ and *H* are multiplied together.

Moreover, a widely utilized conventional tool for measuring the characteristic time of a signal is autocorrelation (Lepri et al., 1994; Schäfer and Kratky, 2008; Zunino et al., 2010b). Autocorrelation involves the cross-correlation of a signal with itself, facilitating the identification of repetitive patterns and the detection of signal periodicity, particularly in the presence of noise. Let χ(*t*) denote the signal, and the continuous autocorrelation *R*_χχ_(τ) at a delay τ is defined as (Zoughi and Boostani, 2010):


$$\begin{array}{l} R_{\chi \chi }\left(\tau \right)=\bar{\chi }\left(-\tau \right)*\chi \left(\tau \right)\\ \;=\int_{-\infty }^{\infty }\chi \left(t+\tau \right)\bar{\chi }\left(t\right)dt\\ \;=\int_{-\infty }^{\infty }\chi \left(t\right)\bar{\chi }\left(t-\tau \right)dt. \end{array}$$

Where $\bar{\chi }$ represents the complex conjugate and * denotes convolution. If the function is real, $\bar{\chi }\left(t\right)=\chi \left(t\right)$. For a wide-sense stationary process, it is defined as (Zoughi and Boostani, 2010):


$$\begin{array}{l} R_{\chi \chi }\left(\tau \right)=E\left[\chi \left(t\right)\bar{\chi }\left(t-\tau \right)\right]. \end{array}$$

iEEG signals are not inherently stationary; however, by considering epochs, stationarity can be achieved (Zoughi and Boostani, 2010). In this study, it is assumed that 68-s samples can be considered stationary epochs.

To test the stationarity of the dataset, an Augmented Dickey Fuller (ADF) test (Mushtaq, 2011; Avramidis et al., 2021) was conducted on all continuous signals within the dataset, using a significance level of 0.05. All signals showed evidence of stationarity. In the W mode, no signals contained NaN values, so all signals were considered stationary when subjected to the test.

**Figure 3:**
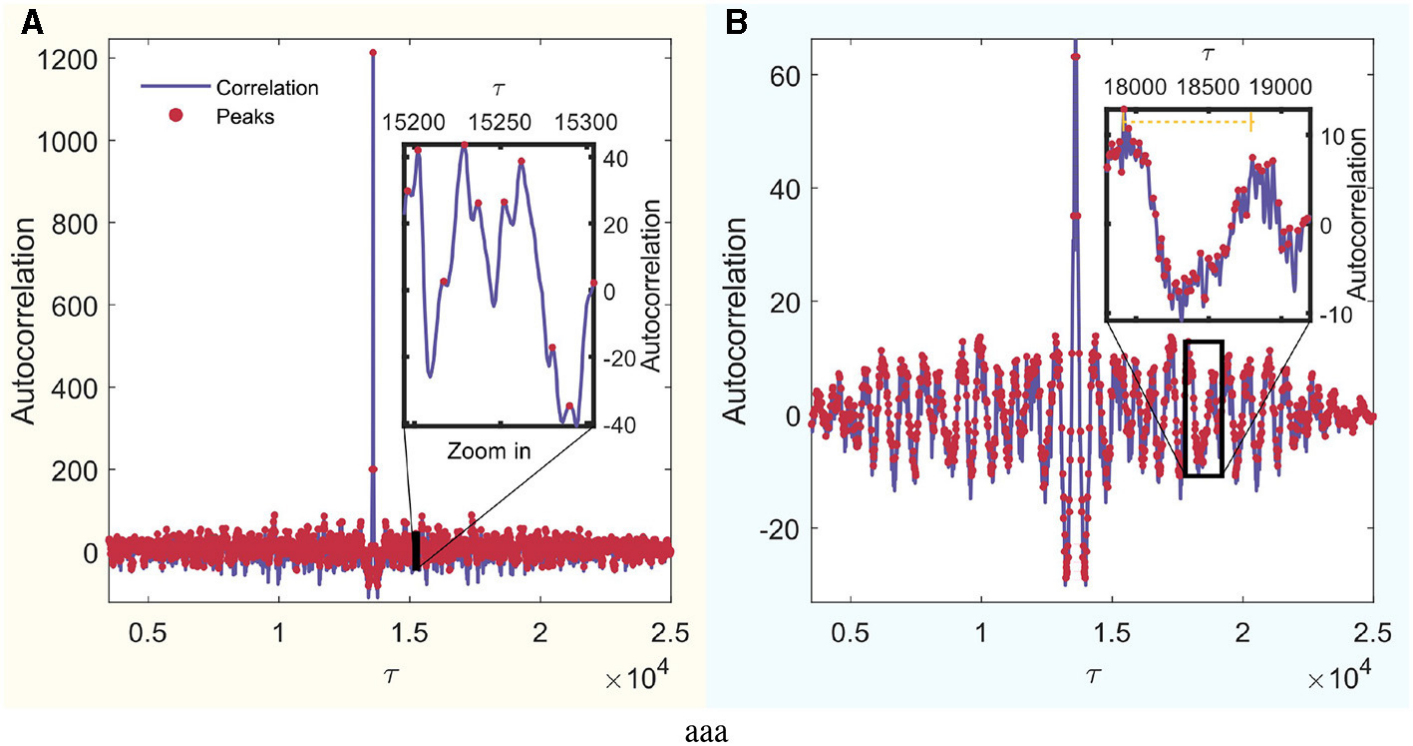
Autocorrelation analysis of a random signal. The x-axis is in units of τ, which can be converted to real time by dividing by the sampling frequency. Identified peaks are highlighted in red, and a detailed zoom-in section is provided for enhanced peak observation. **(A)** Wakefulness with eyes closed (W) mode. **(B)** REM Sleep (R) mode.

Furthermore, to determine τ, the autocorrelation of each signal was calculated, and the distances between the peaks were identified. Subsequently, these distances were averaged to derive a value of τ for each signal. To ascertain τ_*s*_ for each mode, the τ values of all channels were averaged. Figure 3A schematically illustrates this process for an arbitrary signal from the set. In this figure, the autocorrelation is visually represented in blue, and the peaks of local maxima are highlighted with red dots.

When finding the characteristic time (τ_*s*_) of the system by analyzing the peaks of the average statistical complexity of all signals in each state, only the first maximum at τ = 1 is found. Although this method allowed the calculation of τ_*s*_ for single signals and sets of several hundred channels, averaging the complexities of all signals attenuates the peaks, making detection impossible.

To overcome this limitation, autocorrelation is used. This is a technique that measures the similarity between a signal and a delayed version of itself. Analysis of the autocorrelation function helps identify repeating patterns or cycles within the signal. Autocorrelation is applied individually to each signal to find peaks in the autocorrelation function that correspond to the periodicity or characteristic times of the signal. Calculating the average distance between all of these peaks helps to estimate the characteristic short period for each channel (MathWorks, 2024). Finally, the characteristic times are averaged across all channels for each of the four modes. When using these short periods with the Bandt and Pompe (BP) method, smaller-scale causal relationships are taken into account. These short times were preferred in part to preserve this small-scale causality when sampling the signal to create the probability function of ordinal patterns, given the limited length of the signals that ensure stationary epochs to work with.

An example of a REM mode signal is shown in Figure 3B. This is a zoom-in of a section where the signal exhibits a smooth and regular periodicity, like a sinusoidal wave. Its characteristic time surpasses that considered between the sawtooth teeth seen when all local maxima are considered. Nevertheless, this order is on the scale of τ = 800, which, when multiplied by the sampling frequency, yields ~4 s. This constitutes a considerably large separation for constructing the distribution of ordinal patterns when analyzing 60-s signals. Therefore, shorter times were chosen. Analyzing correlations between widely separated regions relative to signal length is beyond the scope of this study. Using τ significantly larger than *D* could result in the loss of significant causal information on small time scales.

**Figure 4:**
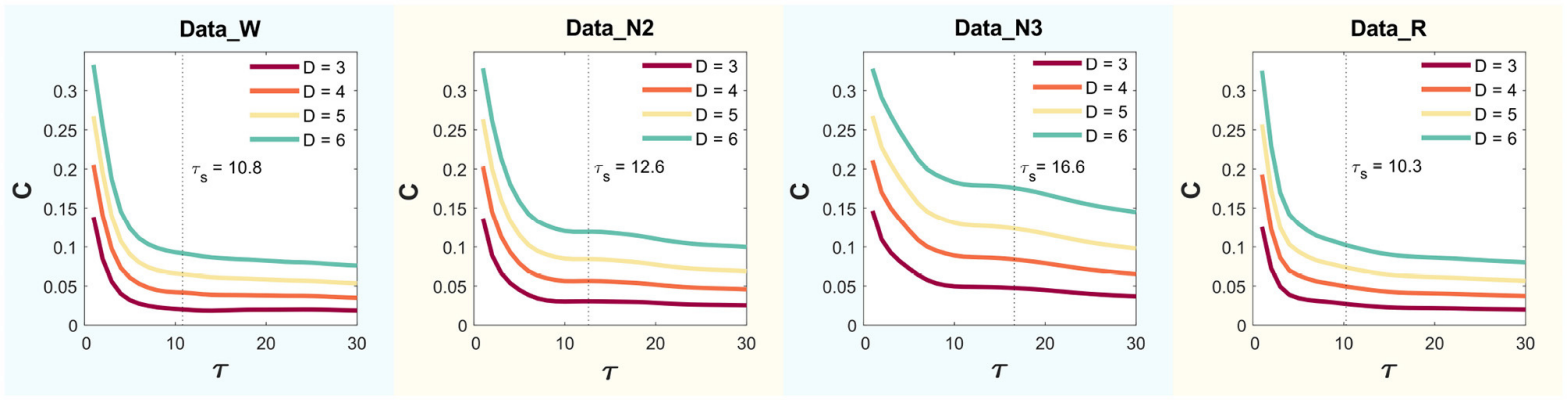
Statistical complexity (*C*) for delay time values τ ranging from 1 to 30 using *D* = 3, 4, 5 and 6.

This yielded values of $\tau_{s}^{W}=10.8$, $\tau_{s}^{N2}=12.6$, $\tau_{s}^{N3}=16.6$, and $\tau_{s}^{R}=10.25$, for Wakefulness with Closed Eyes, non-REM Sleep Stage N2, non-REM Sleep Stage N3, and REM Sleep modes, respectively. Figure 4 presents the MPR complexity curves as functions of τ across dimensions *D* = 3, 4, 5, and 6 for each mode. The corresponding characteristic times τ_*s*_, computed via autocorrelation, are also indicated.

Regarding the Rényi parameter, in this study, quantifiers *H*_*q*_ and *C*_*q*_ were calculated for *q* in the range [0.1, 7] with a step size of 0.01. As a specific case to visually illustrate the differences in mean values of these quantifiers across regions, the value of *q* that maximizes Rényi complexity, calculated analogously to τ, was chosen. For *D* = 6, this resulted in $q_{max}^{W}=1.4$, $q_{max}^{N2}=0.93$, $q_{max}^{N3}=0.81$, and $q_{max}^{R}=1.06$.

### 2.6 Rényi entropy-complexity causality space

The need to introduce the concept of Rényi entropy-complexity causality space (*H*_*q*_ × *C*_*q*_ × *q*) arises from the necessity to distinguish between different curves of Rényi complexity-entropy. These curves are formed by points derived from different parameters *q*, within the same distribution. Sometimes these curves overlap when projected onto a single plane. This is true even though their values differ when compared at certain *q* values.

When computing generalized quantifiers such as the pair *H*_*q*_ and *C*_*q*_, it is necessary to generalize the concept of the entropy-complexity causality plane (*H* × *C*) proposed by Rosso et al. (2007) in order not to lose information in the graphical representation. By introducing the dimension corresponding to the parameter *q*, it is possible to construct a space, which is analogously called the Rényi Entropy-Complexity Causality Space (*H*_*q*_ × *C*_*q*_ × *q*), formed by the superposition of multiple causality planes for successive values of *q*.

The bounds of maximum and minimum complexity can be calculated using the methodology of Martin et al. (2006). These bounds indicate the theoretical upper and lower bounds of complexity and serve as a benchmark for placing different dynamical systems in each *q* plane within the space. They depend on the choice of parameters of the BP *D* and the Rényi parameter *q*.

In this work, the expressions corresponding to Rényi entropy and complexity were modified for the calculation of maximum and minimum complexity bounds in the Ordpy library (Pessa and Ribeiro, 2021)). This library enables the implementation of the BP methodology for Rényi entropy and complexity. Additionally, readers can find the modified bounds functions for Rényi complexity as supplementary material to accompany this Python library.^1^

This space is constructed by stacking multiple causal planes (*H*_*q*_ × *C*_*q*_) within the interval *q*∈{0.5, 0.7, 1, 1.4, 2, 3, 4, 5, 6, 7}, following the approach by Martin et al. (2006). The Logistic Map (*r* = 4), the Hénon Map (*a* = 1.4 and *b* = 0.3), Schuster Map (parameter *z*∈{3/2, 2, 5/2}), white noise, and correlated noise for *k* were represented. Below are the equations of the maps used to generate the temporal series (Pessa and Ribeiro, 2021).

Logistic Map:


$$\begin{array}{l} x_{t+1}=rx_{t}\left(1-x_{t}\right). \end{array}$$

Hénon Map:


$$\{\begin{array}{l} x_{t+1}=1-ax_{t}^{2}+y_{t},\\ y_{t+1}=bx_{t}. \end{array}$$

Schuster Map:


$$\begin{array}{l} x_{t+1}=\left(x_{t}+x_{t}^{z}\right)\;\mathrm{mod}\;1. \end{array}$$

### 2.7 Exploring *f*^−*k*^ behavior across frequency domain: insights from power spectral density

The *f*^−*k*^ behavior of each region under study was explored in the frequency domain by calculating the Power Spectral Density (PSD).

For the PSD estimation, Welch's method was employed on iEEG signals. This involved computing the magnitude of the discrete time Fourier transform for 59 overlapping blocks, each lasting 2*s* with a 1*s* step, and weighted by a Hamming window. Averaging these transformed blocks yielded the spectral density for each channel. To ensure independence from the amplitude of the iEEG signal, the spectral density in each channel was subsequently normalized to total power. These parameter choices were guided by prior work from the developers of the MNI Open iEEG Atlas, as referenced in Frauscher et al. (2018a,b); von Ellenrieder et al. (2020).

The power law coefficient *k* was determined by fitting an exponential function to the data's PSD within the 10–40 Hz range. The lower limit of 10 Hz was selected instead of 1 Hz due to a shoulder between 1 and 10 Hz observed in most channels, as indicated by the atlas authors (Frauscher et al., 2018a). The upper limit of 40 Hz is set by the data's sampling rate. The highest frequency unaffected by anti-aliasing downsampling filters is 80 Hz, but accurate computation of the scale-free spectrum is limited to half that frequency (Frauscher et al., 2018a). Each data channel's PSD was adjusted using the least squares method with the formula *A*·*f*^−*k*^, where *A* represents the amplitude and *k* is the exponent. Subsequently, the parameters from each channel in all regions were averaged individually for males and females. The mean fits and their corresponding coefficient of determination (*R*-square, denoted as *R* below) were plotted on the mean PSD. To compare, a boxplot was generated for males and females, showing the median, interquartile range (IQR), outliers, and a notch representing the 95% confidence interval of the median power law coefficients (*k*) from the fits.

## 3 Results

In this section, the theoretical upper and lower bounds of Rényi complexity for τ = 1 and embedding dimensions *D* = 3, 4, 5, and 6 in space (*H*_*q*_ × *C*_*q*_ × *q*) are presented. Also shown are sections at certain values of *q*, illustrating the position of the classical dynamical systems studied.

Two examples of the implementation of this space are then given: Case 1 involves theoretical simulations of k-noise, while Case 2 involves an application to real experimental data. Embedding dimensions ranging from *D* = 3 to *D* = 6 were employed, with a fixed time delay of τ = 1 for all cases, except in the iEEG time series data where τ = τ_*s*_ was also analyzed. The Rényi parameter varies between values *q*∈[0.1, 7] to compute the quantifiers *H*_*q*_ and *C*_*q*_. The space (*H*_*q*_ × *C*_*q*_ × *q*) and the projections onto the plane (*H*_*q*_ × *C*_*q*_) were plotted for all cases.

In the case of experimental data, time delays of τ = 1 and τ = τ_*s*_ were implemented. Additionally, embedding dimensions of *D* = 3, 4, 5, and 6 were examined. Results for *D* = 6 are presented and compared with the power law exponents obtained for the PSDs of each region across all modes. A schematic representation of the brain has been included, highlighting the differences in the mean values across the analyzed regions for the case of *q* = *q*_*max*_.

### 3.1 Rényi entropy-complexity causality space

**Figure 5:**
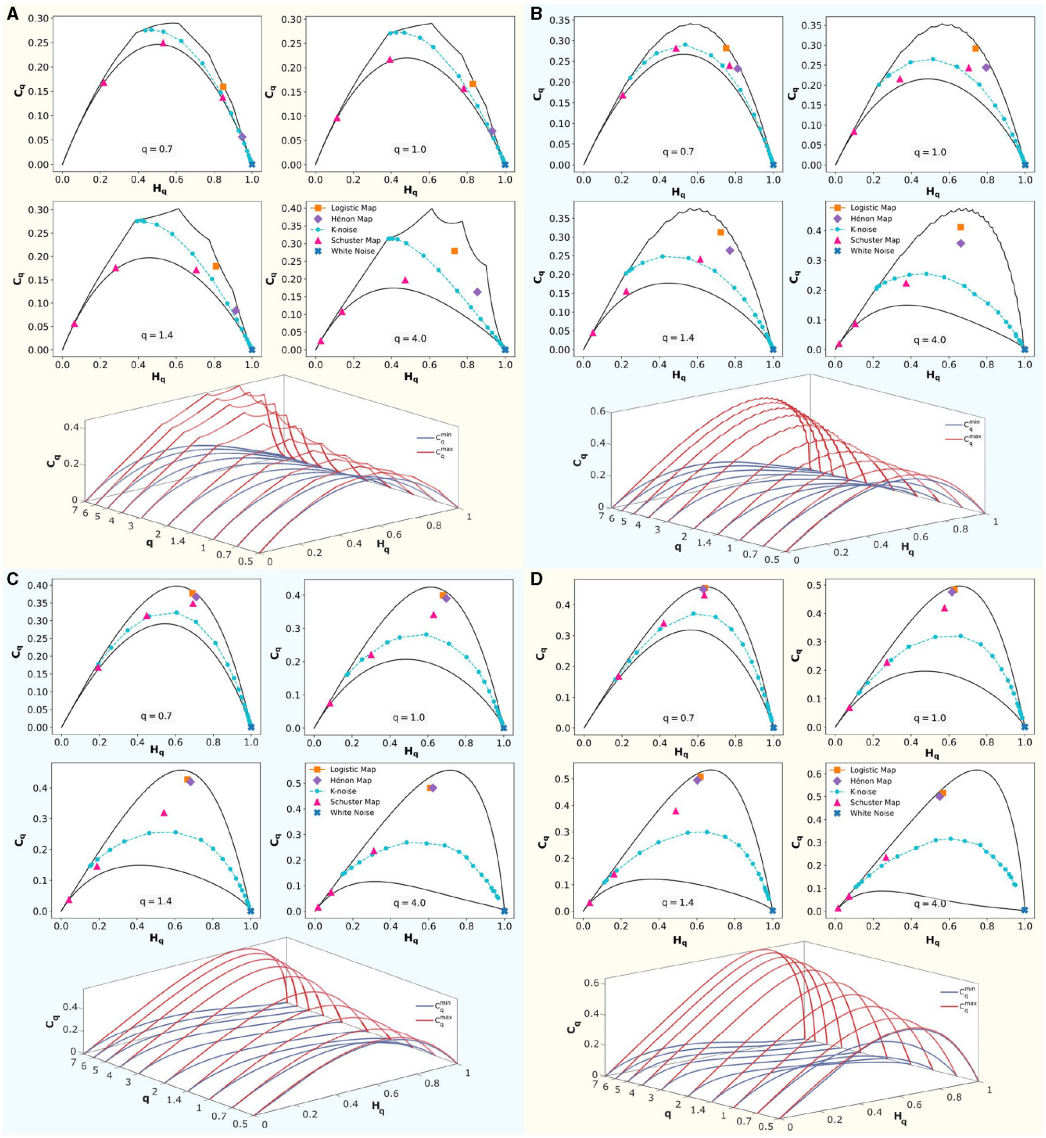
Rényi entropy-complexity causality plane (*H*_*q*_ × *C*_*q*_) for various values of *q*, and Rényi entropy-complexity causality space (*H*_*q*_ × *C*_*q*_ × *q*). For embedding dimensions *D*∈{3, 4, 5, 6} and a delay τ = 1. In each panel for *D*, (*H*_*q*_ × *C*_*q*_) is depicted with its maximum ($C_{q}^{max}$) and minimum complexity ($C_{q}^{min}$) bounds with continuous black lines for Rényi parameter values *q*∈{0.7, 1.0, 1.4, 4.0}. Orange corresponds to the Logistic Map (*r* = 4), violet to the Hénon Map (*a* = 1.4 and *b* = 0.3), and magenta to the Schuster Map (with parameter *z*∈{3/2, 2, 5/2}). Blue represents white noise, while cyan represents correlated noise for *k*∈[1, 5]. Note that *k* values increase from right to left. Below, in each panel, (*H*_*q*_ × *C*_*q*_ × *q*) is displayed by overlaying several planes of parameter *q*∈{0.5, 0.7, 1, 1.4, 2, 3, 4, 5, 6, 7}. The upper limit ($C_{q}^{max}$) is shown in red, and the lower limit ($C_{q}^{min}$) in blue.

In Figure 5, four panels show embedding dimensions *D* = 3, 4, 5, and 6, with a fixed time delay of τ = 1. At the top section of each panel, classical dynamical systems are shown to illustrate their positions in the (*H*_*q*_ × *C*_*q*_) causality planes corresponding to *q*∈0.7, 1, 1.4, 4, presented in reading order. These systems are bounded by the maximum ($C_{q}^{max}$) and minimum complexity ($C_{q}^{min}$) lines, shown as solid black lines. Logistic Map (*r* = 4), the Hénon Map (*a* = 1.4 and *b* = 0.3), the Schuster Map (parameter *z*∈3/2, 2, 5/2), white noise, and correlated noise for *k*∈[1, 5], with increasing values of *k* from right to left, were represented.

Below each panel, the (*H*_*q*_ × *C*_*q*_ × *q*) space is shown schematically. The maximum complexity ($C_{q}^{max}$) is shown in red, and the minimum complexity ($C_{q}^{min}$) is shown in blue. At q = 1, the classical Jensen-Shannon complexity-entropy causal plane (Rosso et al., 2007) is evident. For *q* > 1, $C_{q}^{max}$ increases and $C_{q}^{min}$ decreases, expanding the range of possible values. Conversely, for *q* < 1, these bounds approach each other, reducing the area between the curves. The space (*H*_*q*_×*C*_*q*_×*q*) is defined by the volume formed between these curves as the number of *q* values considered increases.

The position of the systems within the space defined between the bounds depends on the embedding dimension D used. To distinguish system features, one should choose an appropriate τ, optimized according to some criterion (e.g., maximizing complexity), and implement the D that maximizes the differences between the dynamical systems under study. Chaotic systems such as the Hénon map and the logistic map separate at low dimensions and converge as D increases. The same trend is observed for the three implemented cases of the Schuster map. In the case of correlated noise, this behavior depends on the values of k studied.

### 3.2 Case 1: analysis of simulated time series of correlated noise (k-noise)

**Figure 6:**
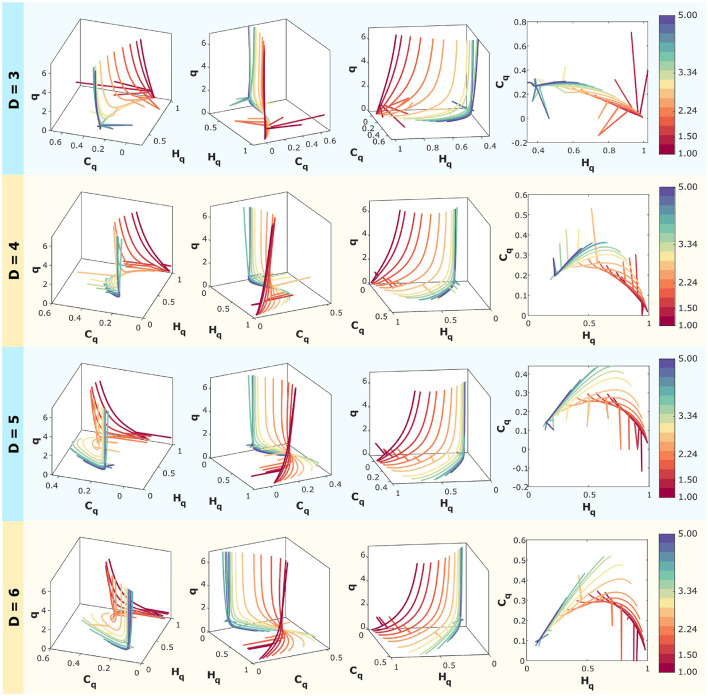
Rényi entropy-complexity causality space for correlated noise for D = 3, D = 4, D = 5, and D = 6 using τ = 1. The temporal series correspond to *f*^−*k*^ with logarithmically spaced values of *k* ranging from 1 to 5. The color scale on the right corresponds to the values of *k* for each curve. For each dimension, three different views of the space (*H*_*q*_ × *C*_*q*_ × *q*) are shown, along with the projection onto the (*H*_*q*_, *C*_*q*_) plane.

In Figure 6, computationally simulated correlated noise (*f*^−*k*^ with *k* ranging from 1 to 5 spaced logarithmically) is analyzed.

Rényi entropy-complexity causality (*H*_*q*_ and *C*_*q*_) calculations have been performed using embedding dimensions of *D* = 3, 4, 5, and 6, with a fixed time delay of τ = 1. This is presented in four panels, one for each dimension. Time series were generated with a length of *M* = 10000, and the Rényi parameter varies within the range *q*∈[0.1, 7].

For each dimension, three different views of the Rényi Entropy-Complexity Causality Space (*H*_*q*_ × *C*_*q*_ × *q*) are presented, with the projection onto the *H*_*q*_ × *C*_*q*_ plane on the right. A graduated color scale is used to represent different values of the exponent *k*.

In the three-dimensional representations, the entropy-complexity curves of the different series do not intersect, allowing the dynamics of the time series with different noise values of *k* to be distinguished in space. In the case of projections onto the plane (*H*_*q*_ × *C*_*q*_), it is more difficult to separate the curves because they touch at points where the values of (*H*_*q*_, *C*_*q*_) are the same, even though they come from different *q* parameters.

Care must be taken when choosing the embedding dimension D, as the behavior in space appears to be opposite to that of the plane, which is obtained by projecting three-dimensional curves onto it. This can be seen especially for the intermediate values analyzed, where the curves open up in space as *D* decreases, but when projected onto the plane, the overlap is greater.

### 3.3 Case 2: analysis of iEEG time series data

This section presents the results of the *H*_*q*_ × *C*_*q*_ × *q* curves for both sexes, considering the standard deviation and the exponents of the fits made to the PSD considering the notches. Emphasis is placed on the differences found in the *H*_*q*_ × *C*_*q*_ × *q* curves, relating them to the differences found in the *k* exponent of the power law (*f*^−*k*^).

**Figure 7:**
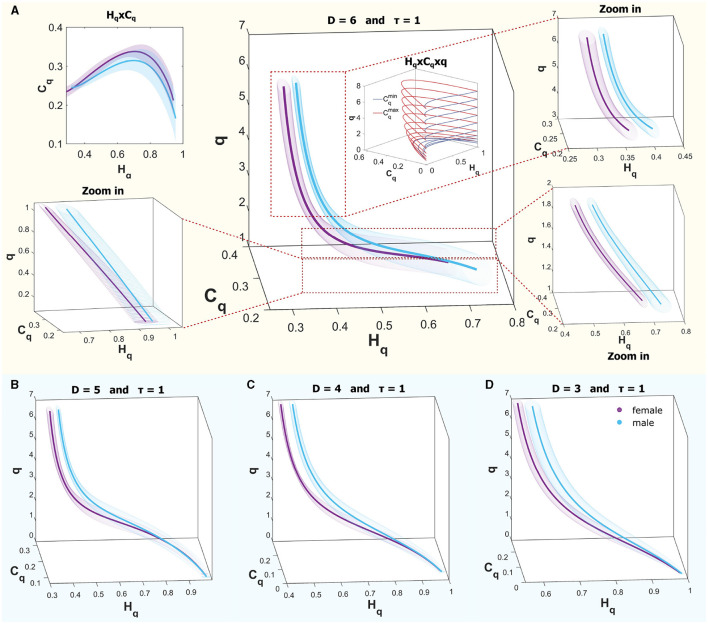
**(A)** Rényi entropy complexity causality space for a single ROI at *D* = 6, τ = 1, illustrating results for both sexes, where the curves are not entirely separated. Theoretical bounds of Rényi complexity are schematically included within the graph. Surrounding this are three zoomed-in sections detailing q-ranges of 0.1–1, 1–2, and 3–7. The top-left inset depicts the projection of the curves onto the complexity-entropy plane. **(B–D)** display entropy-complexity causality spaces for *D* = 5, *D* = 4, and *D* = 3, respectively, across the analyzed q-range [0.1, 7].

Figure 7 provides an illustrative comparison of curves within the causal space (*H*_*q*_ × *C*_*q*_ × *q*) for both sexes, showcasing the ROI of region B for the R mode (REM sleep). The curves within the interval *q* ∈ (0, 7) do not show complete separation; however, for values greater than 1, separation occurs when considering one standard deviation, while they begin to converge as values approach 0. In the upper panel, Figure 7A displays the curves for males (in cyan) and females (in violet) across the full range, with a zoom to three q intervals: 0.1–1, 1–2, and 3–7 for D = 6. A schematic of the theoretical bounds is included in the center, illustrating that for values < 1, the space rapidly contracts, bringing all systems closer together. The upper corner presents the plane (*H*_*q*_ × *C*_*q*_), where the curves almost entirely overlap across the plane when considering deviations. Below, in Panels B–D, the same curves are shown in the space (*H*_*q*_ × *C*_*q*_ × *q*) for D = 5, D = 4, and D = 3, respectively. In all cases, a time delay of τ = 1 was used. It is observed that similar behaviors are found in other dimensions.

**Figure 8:**
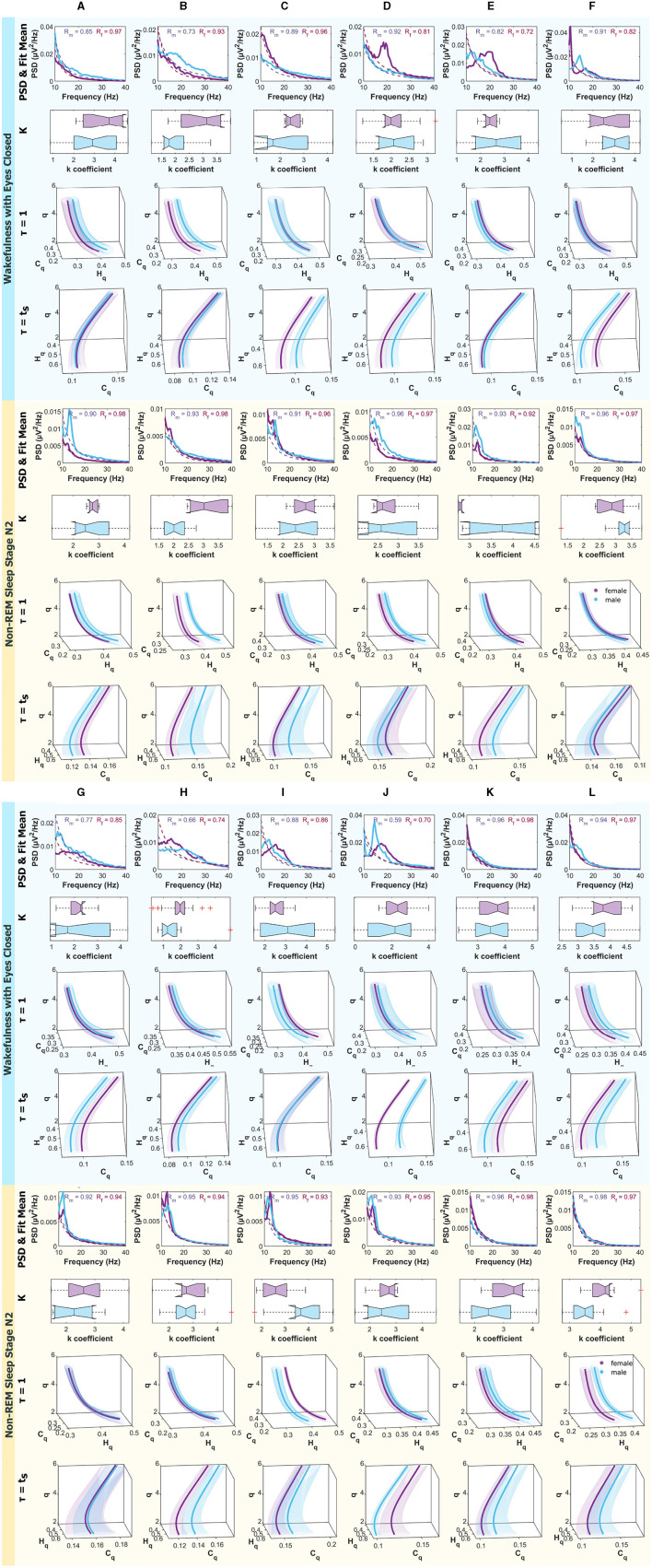
Mean PSD (Power Spectral Density), k-exponent, and the Rényi entropy-complexity causality space (*H*_*q*_ × *C*_*q*_ × *q*). The top panel corresponds to Wakefulness with Closed Eyes, and the bottom one to non-REM Sleep Stage N2. Each column represents an ROI defined in Figure 1. The top row shows the mean PSD (Power Spectral Density) in the 10–40 Hz range with the mean exponential fit represented with dashed lines, the mean R values of the fits are shown above. Below is a boxplot for the power law coefficient k. In the two bottom rows, the (*H*_*q*_ × *C*_*q*_ × *q*) is depicted for τ = 1 and τ = τ_*s*_ using D = 6. Women are represented in violet and men in light blue. The shaded regions correspond to the standard deviation, depicted with ellipses. Each letter represents an ROI defined in Figure 1.

Figure 8 present, from top to bottom, the mean Power Spectral Density (PSD) within the frequency range of 10 − 40*Hz*, along with the mean fitting and R values for males and females. Additionally, boxplots depicting the power law fitting exponents are shown, as well as the curves within the Rényi Entropy Complexity Space for *D* = 6 with delay times of τ = 1 and τ = τ_*s*_.

Results for females are depicted in violet and for males in light blue. The top panel corresponds to the Wakefulness with Closed Eyes state, and the bottom panel to the non-REM Sleep Stage N2. Each column represents an analyzed Region of Interest (ROI) following the convention of Figure 1.

**Appendix Figure A1:**
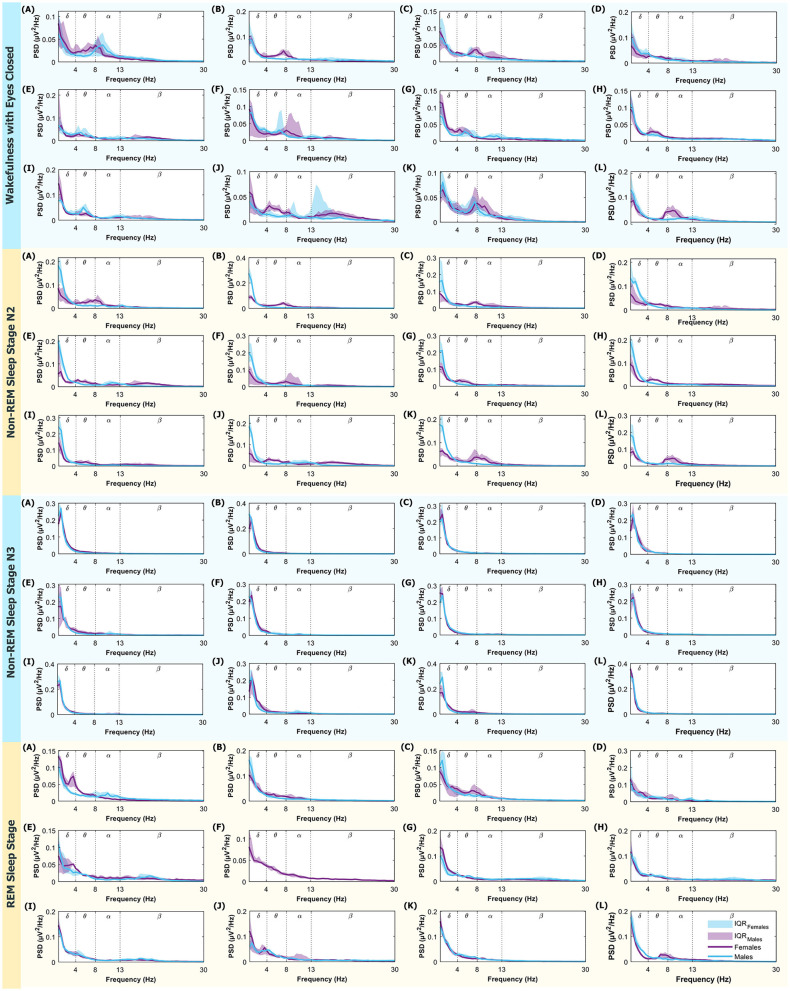
PSD (power spectral density) in the 0.5–30 Hz range. From top to bottom, the modes displayed are: Wakefulness with Closed Eyes, Non-REM Sleep Stage N2, Non-REM Sleep Stage N3, and REM Sleep. Each letter represents an ROI defined in Figure 1. The medians for women are represented in violet and men in light blue. The shaded regions correspond to the IQR (Interquartile Range). Dotted lines mark the boundaries between different brain rhythms.

In the Appendix Figure A1 display, for interested readers, the medians of PSD for males and females are shown, along with the Interquartile Range (IQR) within the frequency range of 0.5 − 30*Hz*, corresponding to the δ, θ, α, and β bands.

**Figure 9:**
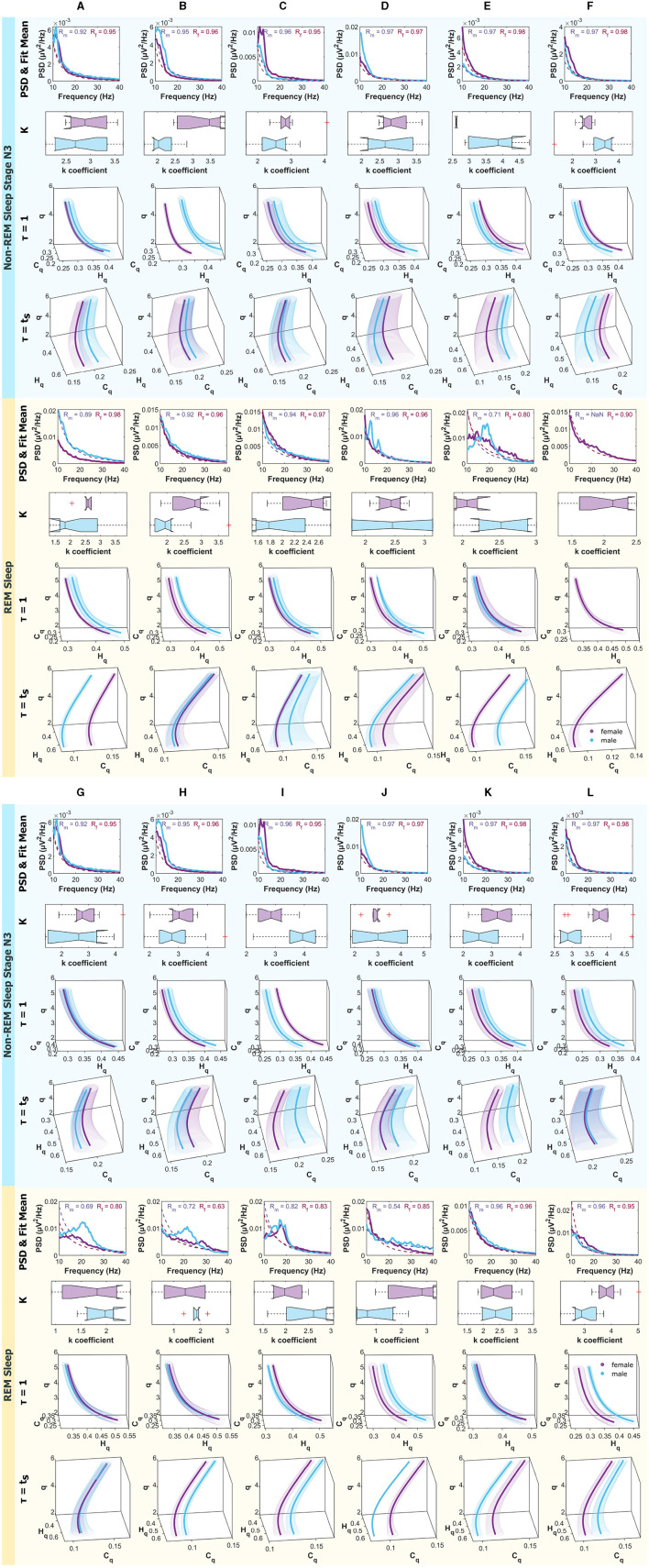
Mean PSD (Power Spectral Density), k-exponent, and the Rényi entropy-complexity causality space (*H*_*q*_ × *C*_*q*_ × *q*). The top panel corresponds to non-REM Sleep Stage N3, and the bottom one to REM Sleep. Each column represents an ROI defined in Figure 1. The top row shows the mean PSD (Power Spectral Density) in the 10–40 Hz range with the mean exponential fit represented with dashed lines, the mean R values of the fits are shown above. Below is a boxplot for the power law coefficient k. In the two bottom rows, the (*H*_*q*_ × *C*_*q*_ × *q*) is depicted for τ = 1 and τ = τ_*s*_ using D = 6. Women are represented in violet and men in light blue. The shaded regions correspond to the standard deviation, depicted with ellipses. Each letter represents an ROI defined in Figure 1.

Figure 9 are analogous to the aforementioned, but the panels correspond to non-REM Sleep Stage N3, and the bottom one to REM Sleep.

Table 2 provides a summary of the sex differences found across all the analyzed regions. The rows show the *k* exponent from the *f*^−*k*^ fit over the PSD, analyzing the notches of the boxplots and the cases of τ = 1 and τ = τ_*s*_, marking the standard deviations of *H*_*q*_ and *C*_*q*_ as shaded ellipses. A “1” indicates that differences were found, while a “0” indicates that no differences were found. Strong sky blue shading highlights regions where differences in exponent were found for both time delays used; lighter shading indicates regions where only one of the τ showed curve separation.

**Table 2:** Summary of differences found between sexes.

**W**	**A**	**B**	**C**	**D**	**E**	**F**	**G**	**H**	**I**	**J**	**K**	**L**
k-exponent	0	1	0	0	0	0	0	1	0	0	0	0
τ = 1	0	1	0	0	0	0	0	0	0	0	0	0
τ = τ_*s*_	0	0	1	0	0	1	1	0	0	1	0	1
**N2**	**A**	**B**	**C**	**D**	**E**	**F**	**G**	**H**	**I**	**J**	**K**	**L**
k-exponent	0	1	0	0	1	0	0	0	1	0	0	1
τ = 1	0	1	0	1	0	0	0	0	1	0	0	1
τ = τ_*s*_	0	1	0	0	1	0	0	0	0	1	0	0
**N3**	**A**	**B**	**C**	**D**	**E**	**F**	**G**	**H**	**I**	**J**	**K**	**L**
k-exponent	0	1	0	0	1	1	0	0	1	0	0	1
τ = 1	0	1	0	1	1	1	0	1	1	0	0	0
τ = τ_*s*_	0	0	0	0	0	1	0	0	1	0	1	0
**R**	**A**	**B**	**C**	**D**	**E**	**F**	**G**	**H**	**I**	**J**	**K**	**L**
k-exponent	1	1	0	0	1		0	0	0	1	0	1
τ = 1	1	1	0	1	0		0	0	1	1	0	1
τ = τ_*s*_	1	0	0	0	1		0	1	1	1	1	1

The columns correspond to the analyzed ROIs, with letter nomenclature referring to Figure 1. A “1” indicates found differences, while “0” indicates no difference.

The cases where differences were found in the exponents and curves simultaneously are detailed below. In the W state, differences were found in τ = 1 in B. In N2, they were found simultaneously in τ = 1 and τ = τ_*s*_ in B, in τ = 1 in I and J, and τ = τ_*s*_ in E. In N3, they were found simultaneously in τ = 1 and τ = τ_*s*_ also in F and I and in τ = 1 in B and E. In R, the greatest number of differences were found, simultaneously in τ = 1 and τ = τ_*s*_ in A, J, and L and in τ = 1 in B, and τ = τ_*s*_ in E.

The ROI that shows the most differences in the curves at the same time as in the exponent is B. On the other hand, in the τ curves, differences were found simultaneously with differences in the *k* exponent with a higher frequency with respect to τ_*s*_, the frequency of occurrence of this event being 12 and 8, respectively.

**Figure 10:**
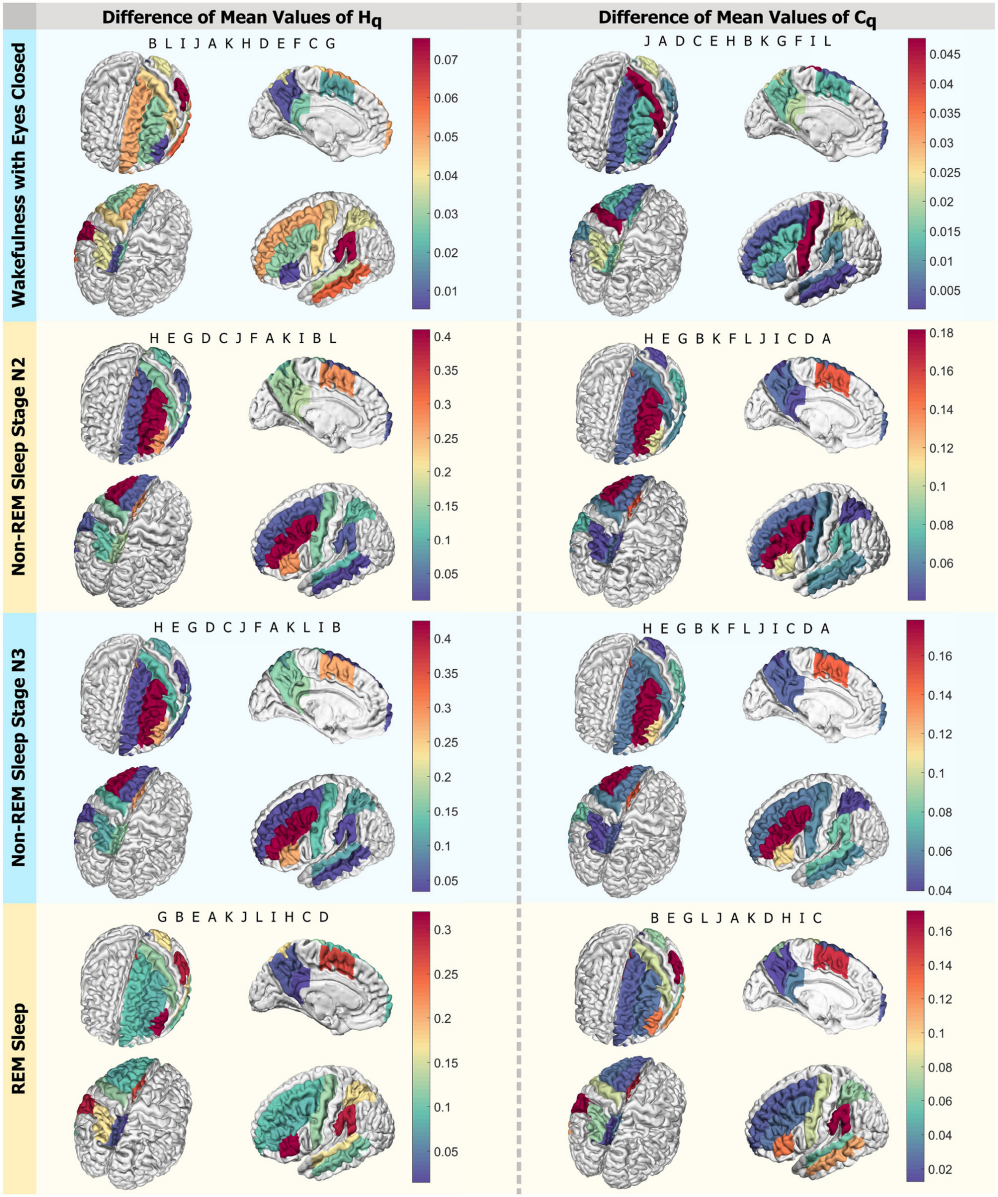
Differences in mean values by region. Scheme of the analyzed ROIs colored according to the intensity of differences in mean values for *q*_max_. The left column displays *H*_*q*_, and the right one presents *C*_*q*_. Each row corresponds to a state: Wakefulness with Closed Eyes, non-REM Sleep Stage N2, non-REM Sleep Stage N3, and REM Sleep. Each panel shows the regions in descending order, with letters following the nomenclature of the ROIs from Figure 1. Each letter represents an ROI defined in Figure 1.

In Figure 10, four panels present a visual scheme illustrating the regions studied using *D* = 6 and *q* = *q*_max_ across the four states investigated: Wakefulness with Closed Eyes, non-REM Sleep Stage N2, non-REM Sleep Stage N3, and REM Sleep from top to bottom. Each panel displays four different views of a brain diagram, color-coded to represent the findings in the regions of interest (ROI) as delineated in Figure 1. The colors indicate the absolute difference in average Rényi entropy and complexity between men and women, ranging from the minimum difference to the maximum (indicated by the scale to the right of each panel). In each instance, the regions are labeled in the order of decreasing differences in the reading direction. For the Wakefulness state, the regions with the most significant differences between men and women in Rényi entropy are B, L, and I, while in complexity, they are J, A, and D. For the N2 and N3 stages of non-REM sleep, the regions with the greatest disparities in entropy and complexity for *q*_max_ in descending order are H, E, and G for both metrics. In the case of REM Sleep, the regions with the most notable differences in entropy are G, B, and E, while for complexity, they are B, E, and G.

## 4 Discussion

This paper proposes a graphical representation of Rényi entropy and complexity in a space that includes the Rényi parameter for all values of *q*. This space is named Rényi Entropy-Complexity Causality Space (*H*_*q*_ × *C*_*q*_ × *q*) following the terminology introduced in Martin et al. (2006). The purpose of this representation is to visually compare curves that exhibit overlaps in 2D when projected onto a single causal plane (*H*_*q*_ × *C*_*q*_). This representation can also be extended to other generalized entropies such as *Tsallis* Ribeiro et al. (2017).

Adjusting the parameter *q* shifts the perspective from treating all events equally (when *q* is close to 0) to focusing on individual probabilities (as in Shannon entropy) or only on the most probable events (when *q* is large). It is like adjusting a mathematical lens to capture different facets of the probability distribution.

The theoretical upper and lower bounds of Rényi complexity for τ = 1 and embedding dimensions *D* = 3, 4, 5, and 6 within the space *H*_*q*_ × *C*_*q*_ × *q* were calculated. The position of classic dynamic systems within cuts of these spaces for the values of *q* = 0.7, 1.0, 1, 4 and 4.0 were represented. In each case, the regions of different dynamic behaviors are shown. Correlated noise separates the regions of chaos (upwards) and stochastic behaviors downwards. Periodic oscillations are located toward the lower left extreme and white noise at the lower right extreme.

This space was constructed for two cases, a theoretical case of simulated correlated noise time series and the application to experimental data. In the first case, the positions of these emulated series of k-noise (*f*^−*k*^) with logarithmically spaced values of *k* from 1 to 5 for τ = 1 and *D* = 3, 4, 5 and 6 were calculated. In all dimensions within the space (*H*_*q*_ × *C*_*q*_ × *q*), the curves separate completely, while the projections on the planes partially overlap. The correlated noise curves of the intermediate k's separate more in the space for D = 3 while the projection of these curves on the plane has a greater overlap.

For the experimental data, signals from the MNI Open iEEG Atlas database (Frauscher et al., 2018a,b; von Ellenrieder et al., 2020) were analyzed. That is, 12 regions were examined, with 5 males and 5 females selected to minimize the differences in mean age between the groups and the deviations in quiet wakefulness with eyes closed (W), non-REM stage N2 (N2), non-REM stage N3 (N3), and REM sleep (R). In each region and mode, the PSDs between (10 − 40*Hz*) were calculated and a fit was made to obtain the k coefficients of the power law (*f*^−*k*^), the results were accumulated in each region and represented with boxplots. A probability distribution was also associated with each time series by the BP method for the embedding dimensions τ = 1 and τ = τ_*s*_ (characteristic time of each mode). In Figures 8, 9, the PSDs, the boxplot of the k's and the curves on the space (*H*_*q*_ × *C*_*q*_ × *q*) are shown. For a better distinction of the scale, they are shown in the range of q values from 2 to 7. Also, because it was intended to explore the general behavior of the system, for large ( q ) values, Rényi entropy focuses on higher-probability events where common patterns dominate. These results are shown for *D* = 6 because this is the dimension in which the differences are more pronounced, although they are also examined in dimensions 3, 4, and 5. An example of how to perform this visual exploration is shown in Figure 7.

Differences were found between sexes in the k exponents and in the curves of the space (*H*_*q*_ × *C*_*q*_ × *q*). The state in which the most differences were found was REM sleep (R) simultaneously. The ROI that shows the most differences in the curves at the same time as in the exponent is B, which corresponds to the region of the Supramarginal gyrus. Greater coincidences were found with the differences in the k exponent when exploring the modes with the classic time delay τ = 1.

The Rényi Entropy-Complexity Causality Space provides a valuable tool for characterizing scale-free dynamics, such as brain dynamics, since it has been suggested that changes in the scaling factor are related to variations in Rényi entropy (Tozzi et al., 2018). In simulated cases, it effectively separates the different components of correlated noise and provides a simple tool to analyze differences between behaviors and characteristics. In applications to experimental data, it is suggested to implement it by plotting the mean value with one or two standard deviations in *H*_*q*_ and *C*_*q*_, forming ellipses, or by plotting the median and making the radii of the ellipses the notches of the boxplot.

In the examined case of sex differences in iEEG, it is necessary to note that the analyzed signals do not only have the scale-free component but also exhibit periodic components, noise, and even chaotic behavior since the time series were used without additional treatment of the atlas. Regarding the limitations, when segmenting by region and attempting to narrow the age range, the statistics are low. Additionally, the data come from regions considered healthy in patients with focal epilepsy, which may introduce another bias. Thus, further studies are needed to confirm these differences. However, the study of these differences in brain activity is of great importance in understanding the differences in unconscious states. At present, despite marked behavioral differences in anesthetic sensitivity, sex differences are not distinguishable in clinically used cortical electroencephalographic recordings (Wasilczuk et al., 2024). That is, the exploration of alternative methods for the evaluation of differences is of great importance.

In future work, the intention is to use this tool to analyze an additional data set that provides us with a segmentation into classes with a larger number of individuals. During this exploration, the focus of the forthcoming work is on the development and application of quantitative methods that can allow us to make a more rigorous and accurate comparison of the experimental data curves. In addition, it is hoped that the results will motivate the development of easy-to-implement tools that facilitate the detection and understanding of scale-free phenomena in the clinical setting. These tools could have a significant impact on the understanding and treatment of various medical conditions, providing new perspectives for research and clinical practice.

## Data availability statement

The original contributions presented in the study are publicly available. This data can be found here: https://github.com/Gisandio/Renyi-Entropy-Complexity-Causality-Space.

## Author contributions

NG: Conceptualization, Data curation, Formal analysis, Investigation, Methodology, Project administration, Resources, Software, Validation, Visualization, Writing—original draft, Writing—review & editing. FM: Conceptualization, Data curation, Formal analysis, Funding acquisition, Investigation, Methodology, Project administration, Resources, Software, Supervision, Validation, Visualization, Writing—original draft, Writing—review & editing.

## Footnotes

^1^ https://github.com/Gisandio/Renyi-Entropy-Complexity-Causality-Space
